# Molecular Simulations of Hydrogen Sorption in Semicrystalline High-Density Polyethylene: The Impact of the Surface Fraction of Tie-Chains

**DOI:** 10.1021/acs.jpcb.3c07705

**Published:** 2024-03-07

**Authors:** Omar Atiq, Eleonora Ricci, Marco Giacinti Baschetti, Maria Grazia De Angelis

**Affiliations:** † Institute for Materials and Processes, School of Engineering, University of Edinburgh, Sanderson Building, Robert Stevenson Road, Scotland EH9 3FB, U.K.; ‡ Department of Civil, Chemical, Environmental and Material Engineering, (DICAM), Alma Mater Studiorum − Università di Bologna, via Terracini 28, Bologna 40131, Italy; § DPI, P.O. Box 902, Eindhoven 5600 AX, The Netherlands

## Abstract

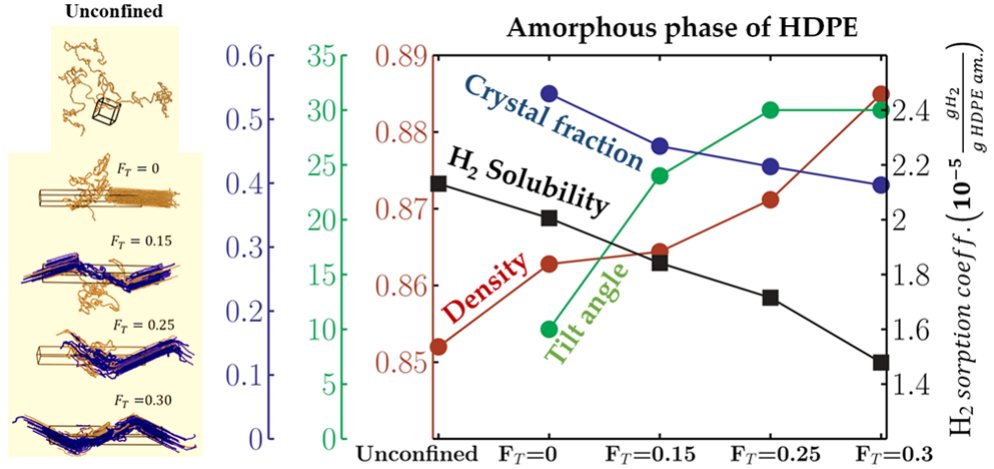

The modeling of the barrier properties of semicrystalline polymers has gained interest following the possible application of such materials as protective liners for the safe supply of pressurized hydrogen. The mass transport in such systems is intimately related to the complex intercalation between the crystal and amorphous phases, which was approached in this work through an all-atom representation of high-density polyethylene structures with a tailored fraction of amorphous–crystalline connections (tie-chains). Simulations of the polymer pressure–volume–temperature data and hydrogen sorption were performed by means of molecular dynamics and the Widom test particle insertion method. The discretization of the simulation domains of the semicrystalline structures allowed us to obtain profiles of density, degree of order, and gas solubility. The results indicated that the gas sorption in the crystalline regions is negligible and that the confinement of the amorphous phase between crystals induces a significant increase in density and a drop in the sorption capacity, even in the absence of tie-chains. Adding ties between the crystal and the amorphous phase results in further densification, an increase of the lamella tilt angle, and a decrease in the degree of crystallinity and hydrogen sorption coefficient, in agreement with several literature references.

## 1 Introduction

The use of semicrystalline polymers has become prevalent in various industries and continues to drive advancements and open new possibilities in numerous fields. Besides the polymer chemical architecture and molecular weight distribution, the control and optimization of the cooling rate, annealing temperature, applied stress, and additive concentration during the crystallization allow us to tailor specific crystallite morphologies and intercalation with the amorphous matrix, thus obtaining materials with finely tuned macroscopic properties. Semicrystalline polymers, in fact, exhibit excellent thermal stability and mechanical and chemical resistance, as well as good optical and electrical properties. Moreover, they are widely used as packaging materials due to their low gas and vapor permeability and ease of processing.^1−3^

Semicrystalline polymers thus naturally became one of the best candidates in new applications created by the advent of the green economy and the shift toward new energy sources. In particular, the current global endeavor toward the transition to a hydrogen-based economy has raised the demand for non-metal materials which could be applied as liners for high-pressure (up to 70 MPa) gas storage tanks (type IV) and pipelines, ensuring minimum leakage and preventing blistering.^4−6^ The lightness and excellent gas barrier performance of semicrystalline polymers make them particularly suited for such applications. However, experimental gas transport data are still scarce, especially for the case of hydrogen, which is challenging to measure.

The permeability of gases across dense materials can be evaluated via the solution-diffusion model as the product of the sorption and diffusion coefficients, which are both strongly affected by the presence of the crystallites.^7−10^ The experimental evidence suggests that the solubility coefficient is primarily reduced because the crystals do not sorb. In addition, the confined amorphous matrix exhibits a lower sorption capacity compared to that in its corresponding unconfined state, and that is usually ascribed to the perturbation induced by the crystallites. On the other hand, the penetrant diffusivity coefficient drops because of the reduced free volume in the constrained amorphous phase and the additional tortuosity induced by the impermeable crystalline domains, which act as physical obstacles hampering the free path of the molecules. It is crucial for the optimization of such materials to improve the knowledge of the interplay between the crystal and amorphous phases.

The crystal–amorphous interface, in fact, is characterized by a variety of chain populations:

i *loops*: chains that exit the crystal lamella and re-enter it in an adjacent position (tight folds) or after exploring the amorphous phase (loose loops).
ii *tails*: chains exiting the crystal phase and ending in the amorphous phase.
iii *floating chains*: free chains not connected to any crystal.
iv *loop entanglements*: entangled loops belonging to different crystallites.
v *bridges*: chains linking two different lamellae that become elastically active “*tie-chains*” when the distance they span is comparable with the length of the amorphous stack.

Tie-chains and loop entanglements are widely recognized as the fundamental topological families when studying semicrystalline polymers.^11−14^ Such molecular connections are responsible for the transmission of the applied stress among crystallites; without their presence, these would be held together just by weak non-covalent interactions, thus leading to brittle behavior. Several studies, therefore, addressed the evaluation of their content using both statistical and experimental approaches.^13,15−18^ Tie-chains and entanglements are also believed to play a significant role in constraining the confined amorphous matrix, which exhibits an increased density and, ultimately, a reduced penetrant solubility.

A variety of models addressed the evaluation of fluid solubility in semicrystalline polymers.^19^ Several works used equations of state (lattice fluid theory or SAFT) of a fully amorphous phase and implicitly accounted for the crystal effect by relying on the Michael & Hausslein theory.^20^ This strategy consists of adding an elastic contribution to the chemical potential, which is dependent on the fraction of elastically effective chains parameter *f* (usually regarded as the fraction of tie-chains).^11,21−24^ Other modeling strategies mimic the overall action of the crystal phase by introducing a fictitious additional pressure, namely, the constraint pressure parameter *p*_c_, to the gas phase pressure.^25−29^ Both modeling strategies require, in most cases, the fitting of the parameter incorporating the crystal effect and hence exhibit limited predictive ability and transferability.

The aim of the present work is to provide a direct prediction of hydrogen sorption coefficients in semicrystalline high-density polyethylene (HDPE), one of the barrier polymers with the best cost/performance trade-off, using molecular dynamics (MD) simulations of an atomistic molecular model in which the crystal–amorphous interface is explicitly reproduced. In particular, the impact of the extent of amorphous–crystal connection, quantified by the surface fraction of tie-chains, on the volumetric properties, orientation, and sorption capacity of the amorphous regions was investigated for several simulated structures.

## 2 Methods

Molecular structures of HDPE were modeled in an all-atom representation, while hydrogen was represented as an uncharged diatomic molecule with two Lennard-Jones (L-J) sites. The PCFF force field was selected for the description of the intra- and intermolecular interactions of HDPE:^30^ it is a class 2 force field that uses a 9–6 L-J pair potential for describing Van der Waals interactions (eq 1), while electrostatic interactions are computed according to Coulomb’s law (eq 2).
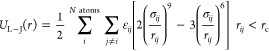
1
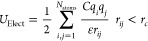
2

**Figure 1:**
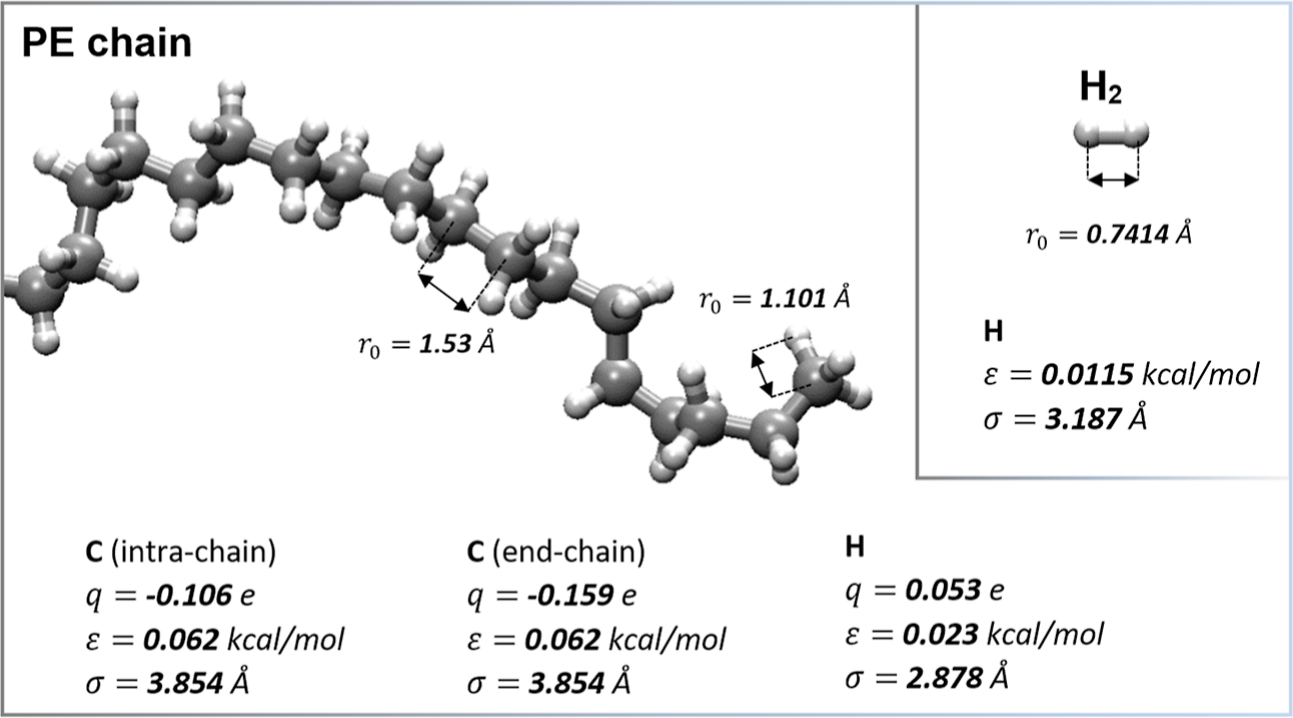
Polyethylene and hydrogen force-field parameters used in this work: PE (PCFF^29,30^) and H_2_ (IFF 9–6 set A^31^).

For the H_2_ molecular model, L-J parameters were coherently taken from a literature work which optimized the latter on pure gas experimental data using the L-J 9–6 functional form.^31^ For the sake of conciseness, only the main parameters involved in the polymer and hydrogen modeling are displayed in Figure 1; the PCFF full functional form and its complete set of parameters can be found in previous works.^29,30^

The choice of an all-atom representation instead of a united atom (UA) one, such as the one used by the TraPPE force field,^32^ is motivated by the demonstrated ability of the PCFF force field to reproduce the polymer pressure–volume–temperature (P–V–T) data of HDPE.^29^ In addition, the small interstices between the polymer atoms, whose shape cannot be properly reproduced by an UA model, might represent an essential contribution for capturing H_2_ solubility through the Widom test particle insertion method.^33^ MD simulations of UA PE using the TraPPE force field may be preferred in case there is the need to simulate the polymer crystallization process in a reasonable computational time,^34,35^ an objective which was beyond the scope of this study.

In the present work, all-atom semicrystalline HDPE structures with tailored surface fractions of tie-chains were built in steps. First, the molecular structure of the crystal phase was constructed and validated, and then it was intercalated with an equally validated amorphous counterpart. MD simulations were performed using LAMMPS,^36^ with van der Waals and electrostatic interactions computed using the *lj/class2/coul/long* command, setting a cutoff of 12 Å; L-J mixed pair coefficients were computed using a sixth power mixing rule (eq 3).
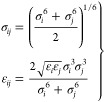
3

van der Waals interactions outside the cutoff were taken into account by applying tail corrections, whereas long-range electrostatics were computed in *k*-space using a particle–particle particle–mesh solver.^37^ Nonbonded interactions between atoms separated by less than three consecutive bonds were excluded from the calculation.

### 2.1 Unconfined Amorphous Phase Model and Simulations

The unconfined amorphous phase was modeled using three HDPE chains of 443 monomers (*M*_w_ = 12429.57 g/mol). Although such a molecular weight is lower compared to that of the typical commercial ones for HDPE, simulations have shown that this value is sufficient to reproduce the experimental volumetric properties of a higher molecular weight polyethylene (126000 g/mol).^29^ Therefore, since the sorption coefficient is mainly dependent on density, the use of longer chains, which require significantly longer equilibration times, was deemed not necessary. The initial configuration was created by inserting the chains simultaneously using the *Amorphous Builder* tool available in the *MAPS Scienomics* software.^38^ The initial cubic simulation box had a side length of 42.617 Å, and 3D periodic boundary conditions were applied. Simulations were performed to reproduce the amorphous polymer P–V–T data from the melt state to ambient conditions. After a *Geometry Optimization* run to remove atom overlaps, the initial configuration was simulated in the *NPT* ensemble at *T* = 473 K and *p* = 0.1 MPa for 20 ns. The initial velocity ensemble was assigned according to a Gaussian distribution, which reproduced the selected temperature with zero linear and angular momentum, while the pressure tensor diagonal components were coupled together (*iso* command). The Nosè–Hoover thermostat and barostat were used for the temperature and pressure control during the simulation with damping factors of 10 and 350 fs, respectively. Newton’s equations of motion were integrated using the velocity Verlet algorithm with a time step of 1 fs; configurations were saved every 10 ps. At the end of the equilibration at 473 K, the system was cooled down to 293 K, fixing temperature steps of 10 K with a cooling rate of 1 K/ns and performing a 20 ns equilibration at constant temperature after each ramp. The P–V–T data of the unconfined amorphous phase at 0.1 MPa were finally extracted by averaging the simulated temperature and volume during each equilibration stage.

### 2.2 Crystal Phase Construction and Simulations

As previously mentioned, the polyethylene crystal phase structure was built a priori to bypass the computational effort associated with the direct simulation of the crystallization and for tailoring its degree of intercalation with the amorphous phase by tuning the area fraction of tie-chains. To this end, the crystal orthorhombic unit cell was created using the *Crystal Builder* tool provided by *MAPS*, considering the experimental lattice parameters reported in the literature,^39^ namely: *l*_*x*_ = 7.397 Å, *l*_*y*_ = 4.935 Å, and *l*_*z*_ = 2.546 Å (which resulted from crystallization upon slow cooling from the melt) and the atoms fractional coordinates.^40^ Starting from the primitive unit cell, crystalline lamellar PE structures differing in the length and number of packed aligned chains were built according to the following steps:

1 Multiplication of the crystal unit cell along the three directions (bulk crystal).
2 Removal of carbon atoms on the bottom layer (break the periodicity along the *z*-axis) and of all the hydrogen atoms.
3 Formation of folded aligned chains with tight adjacent re-entries by creating alternating C–C bonds on the upper and lower surfaces along the *x*-axis direction.
4 Reassignment of all hydrogen atoms and quick *Geometry Optimization* run (100 steps) to optimize the established fold conformation.

**Figure 2:**
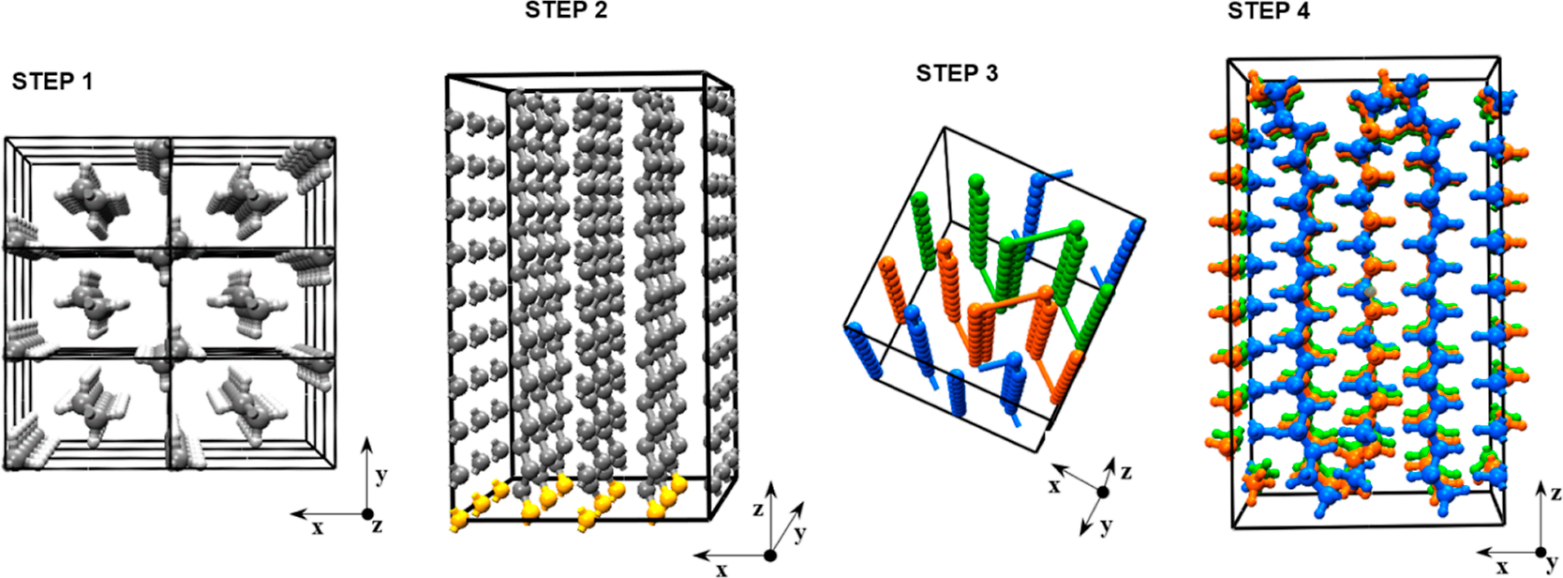
Illustration of the steps involved in the construction of the crystalline PE lamellae: STEP 1, propagation of the crystal unit cell along the three directions; STEP 2, removal of carbon atoms on the bottom layer and of all the hydrogen atoms; STEP 3, formation of folded aligned chains with adjacent re-entries; and STEP 4, reassignment of all hydrogen atoms and geometry optimization.

For the sake of clarity, a visual rendering of the strategy is illustrated in Figure 2.

The details of the whole set of crystalline lamellae that were built according to the aforementioned procedure are summarized in Table 1 in terms of dimensions, number of folded chains, and chain length (monomers). In this table, *L*_*x*_, *L*_*y*_, and *L*_*z*_ represent, respectively, the edge length of the lamella relative to (*l*_*x*_×), (*l*_*y*_×), and (*l*_*z*_×) multiplication of the primitive unit cell edges.

**Table 1:** Properties of PE Crystalline Lamellae Built in This Work with the Procedure Reported in Figure 2: Dimensions (Number of Repetitions of the Primitive Unit Cell), Thickness, Number, and Length of Packed Chains

crystal structure	*L*_*x*_ (*l*_*x*_ ×) [Å]	*l*_*y*_ (*l*_*y*_ ×) [Å]	*L*_*z*_ (*l*_y_ ×) [Å]	thickness [*nm*]	chain length [*monomers*]	N chains
lamella_4 nm_8 × 124	29.588 (4)	39.480 (8)	40.736 (16)	4.07	124	8
lamella_8 nm_4 × 252	29.588 (4)	19.740 (4)	81.472 (32)	8.15	252	4
lamella_8 nm_8 × 504	59.176 (8)	39.480 (8)	81.472 (32)	8.15	504	8
lamella_16 nm_2 × 508	14.794 (2)	19.740 (4)	162.944 (64)	16.29	508	2
lamella_16 nm_6 × 508	29.588 (4)	29.610 (6)	162.944 (64)	16.29	508	6
lamella_16 nm_4 × 635	29.588 (4)	24.675 (5)	162.944 (64)	16.29	635	4

As for the case of the amorphous phase, MD simulations were carried out to evaluate the P–V–T data of the lamellar structures and their dependence on the different features of the lamellae. Simulations were carried out applying the same simulation setting previously mentioned, except for the pressure tensor diagonal components that were set as uncoupled (“*aniso*” command) due to the anisotropic nature of the structure. The lamellae obtained in the last step described in Figure 2 were equilibrated first in the *NPT* ensemble at 298 K and 0.1 MPa for 20 ns. The resulting equilibrated structures at ambient conditions were then heated up to 353 K using temperature steps (Δ*T*) of 10 K (5 K for the first step) according to the following recursive path

- *NPT* temperature ramp from *T*_*i*_ to *T*_*i*_ + Δ*T*, *p* = 0.1 MPa (200 ps)
- *NVT* at *T*_*i*_ + Δ*T* (50 ps)
- *NPT* at *T*_*i*_ + Δ*T* and *p* = 0.1 MPa (250 ps)

The simulated P–V–T data of the lamellae were finally extracted by averaging the simulated temperature and volume at stage 3. The comparison of the latter against the experimental references guided the selection of the molecular model of the crystal phase to be incorporated in the semicrystalline structure. In particular, the lamella having a thickness of 16 nm formed by 4 chains of 635 monomers was selected, as explained in the Results section.

### 2.3 Semicrystalline Structure Construction and Simulation

Once the amorphous and crystal phase molecular models were tested, semicrystalline structures with different surface fractions of tie-chains were created. Structure A was built by simply assembling a bilayer, thus placing an amorphous phase cell over the selected crystalline lamella cell (initial configuration from step 4) through the *Interface Builder* tool available in MAPS. The simulation cell basal plane of the amorphous phase, indeed, was set equal to the crystal one, whereas the cell height, corresponding to the interlamellar stack thickness (*z*_am.stack_), was chosen to guarantee an initial amorphous phase density of 0.852 g/cm^3^, equal to the theoretical unconfined value at ambient conditions (eq 4). The latter comes as an output of the MD simulations introduced in 2.1 and will be reported in the relative Results section. The *L*_*x*_ and *L*_*y*_ edge lengths are reported in Table 1.
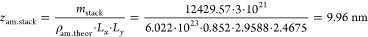
4

Structure A clearly represented only the bare confinement of the amorphous phase between crystalline lamellae since no direct connection between the two phases had been established. To introduce this effect, three additional structures with a progressively higher surface fraction of ties were built, by connecting the crystal upper and lower surfaces with a chosen number of tie-chains and finally adjusting the density of the amorphous stack to the theoretically unconfined one by adding unconnected amorphous chains.

The surface fraction of tie-chains, *F*_T_, was defined as the ratio between the number of established tie-chains and the total number of stems on the lamella cross-section. Assuming that the cross-sectional area of a single stem is equal to the one occupied by a tie-chain emerging from the crystal lamella, the latter approximates well the area fraction that ties occupy in the crystal–amorphous cross-section:
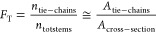
5

A number of ties equal to 6, 10, and 14 were used for the construction of structures B, C, and D, respectively, corresponding, on a total of 40 stems, to *F*_T_ equal to 0.15, 0.25, and 0.35. Such values are within the range of experimental estimates of the area fraction of tie molecules based on mechanical analysis, such as slow crack growth (*F*_T_ = 0.007–0.23^41^), or on statistical approaches (*F*_T_ = 0.145–0.51^16,17^).

It is worth highlighting that, before starting the simulation, the four semicrystalline structures shared the same initial degree of crystallinity, amorphous stack thickness, and density. Moreover, the initial lamella tilt angle θ_tilt_ (the angle formed between the lamella chain direction and the *z*-axis), by construction default (see Figure 2), was equal to zero for all the structures.

During simulations, a first path was aimed at obtaining a reliable equilibrated configuration at ambient conditions for all the semicrystalline structures, while a second step was addressed at evaluating the P–V–T data, starting from the equilibrated configurations. The first simulation pathway comprised a preliminary *Geometry Optimization* of the structure configuration, followed by a heating stage and equilibration at high temperatures:

- *NPT* temperature ramp from 298 to 473 K, *p* = 0.1 MPa (2 ns)
- *NPT* at *T* = 473 K and *p* = 0.1 MPa (5 ns)

This stage, too short to induce melting of the crystal phase, has the aim to relax the pristine crystalline lamella by enhancing chain sliding and loose fold formation as well as loosening the established tie-chains, which, by construction, were fully stretched in the initial conformation.

The configurations resulting from the latter stage were then compressed at a constant temperature and subsequently equilibrated at high temperature and pressure:

- *NPT* pressure ramp from 0.1 to 10 MPa, *T* = 473 K (2 ns)
- *NPT* at *p* = 10 MPa and *T* = 473 K (2.75 ns)

This stage was performed to further drive the interpenetration of the crystal and amorphous phases to enhance a smoother crystal–amorphous transition. Subsequently, the resulting configurations were cooled stepwise to 298 K and decompressed to 0.1 MPa using temperature steps Δ*T* of 20 K (15 K for the last step) and pressure steps Δ*p* of 1 MPa (0.9 MPa for the last step) according to the following sequential scheme:

1 *NPT* temperature ramp from *T*_*i*_ to *T*_*i*_—Δ*T* and pressure ramp from *p*_*i*_ to *p*_*i*_—Δ*p* (200 ps)
2 *NVT* at *T*_*i*_ -Δ*T* (50 ps)
3 *NPT* equilibration at *T*_*i*_ -Δ*T* and *p*_*i*_ -Δ*p* (750 ps)

Finally, the resulting configurations at *T* = 298 K and *p* = 0.1 MPa were well-equilibrated through a longer *NPT* run (40 ns), thus leading to the final structures at ambient conditions.

The second simulation pathway split into two specular routes in which the last configuration of the equilibrated structures at ambient conditions was, respectively, cooled down to 283 K and heated up to 313 K at a rate of 1.5 K/ns and subsequently equilibrated at constant temperature for 20 ns. The collection of P–V–T data on semicrystalline structures was carried out by averaging the simulated temperatures and volumes in the last 10 ns of the equilibration stages at 283, 298, and 313 K for each structure.

### 2.4 Sorption Coefficient Evaluation

Hydrogen sorption coefficients in the theoretical unconfined amorphous matrix and in the semicrystalline structures were evaluated by applying the Widom test particle insertion method^42^ on the equilibrated trajectories at 283, 298, and 313 K. In the *NPT* ensemble, the excess chemical potential of the gas molecule (*i*) in the polymer system (μ_*i*_^*ex*^) can be calculated by estimating the change in internal energy triggered by the insertion of the molecule according to the relation
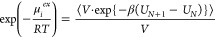
6

where *U*_*N*_ is the potential energy of a given polymer configuration comprising *N* atoms, *U*_*N*+1_ is the potential energy of the polymer configuration plus the randomly inserted molecule, β is 1/*k*_B_*T*, *k*_*B*_ is the Boltzmann constant, *R* is the ideal gas constant, and *V* is the polymer volume. The average is calculated over all of the polymer configurations in use and the total performed insertions. For both the unconfined amorphous and semicrystalline structures, the last 1000 frames of each equilibration stage (corresponding to the last 10 ns) at 283, 298, and 313 K were collected and tested with 2000 random insertions of hydrogen; therefore, a total of 2 × 10^6^ random insertions were performed for each evaluation. The latter was enough to reach convergence of the test, which was identified by the stabilization of the running average of the term exp{−β(*U*_*N*+1_–*U*_*N*_)} to a plateau value.

From the excess chemical potential at infinite dilution, the mass fraction-based Henry’s law constant was estimated according to the following expression:
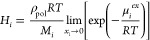
7

where ρ_pol_ is the polymer density and *M*_*i*_ is the gas molecule molecular weight. The mass-fraction-based sorption coefficient, *S*_*i*_, was evaluated as the reciprocal of *H*_*i*_.

#### 2.4.1 Evaluation of Density, Orientation, and Sorption Coefficient Spatial Profiles

A deeper understanding of the gas sorption capacity dependence on the crystal phase content and interaction with the confined amorphous matrix may be obtained by relating the polymer local features and the corresponding gas sorption coefficient. Therefore, for the semicrystalline structures, the frames were discretized along the *z*-axis (the only relevant in terms of crystal–amorphous transition) in 60 slices; the mass density ρ(*z*_*k*_) and bond orientation order parameter *P*_2_ (*z*_*k*_) were computed for each slice *z*_*k*_.

The bond-order orientation parameter was defined according to the following relation:^43,44^
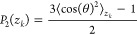
8where θ is the angle formed by a given C–C–C bond chord (vector connecting the centers of two consecutive bonds) and the *z*-axis, while ⟨ ⟩ *z*_*k*_ is the average made over all of the chords that fall within a given slice *z*_*k*_. *P*_2_ tends to 1 if there is alignment of the chains along the chosen axis (crystal domain) and tends to 0 if there is no predominant chain orientation (amorphous phase), thus allowing the identification of the phase boundaries. Finally, the Widom test postprocessing was adapted to the discretized simulation box volume by sorting the performed insertions according to their slice, providing an evaluation of μ_*i*_^*ex*^ and *H*_*i*_ along *z:*
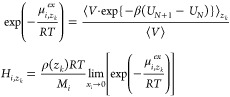
9

Moreover, the drastic change of Henry’s law constant due to the crystal–amorphous transition allowed for the estimation of the confined amorphous phase boundaries [*z*_–_^*am*^, *z*_+_^*am*^]. Based on these, the crystalline and confined amorphous densities were extracted. The mass degree of crystallinity of the structure was then estimated by applying the additivity of the volumes of the crystal and confined amorphous phases (volume balance):
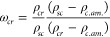
10where ρ_*sc*_, ρ_*cr*_, and ρ_*c.am.*_ are the simulated semicrystalline, crystal, and confined amorphous densities, respectively. Finally, the sorption coefficient within the solely confined amorphous phase was estimated by accounting for the insertions that fell within the interval [*z*_–_^*am*^, *z*_+_^*am*^]:
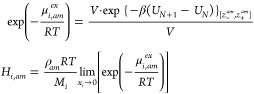
11

Such elaboration was necessary to accurately identify the effect of tie-chains on the sorption capacity of the amorphous phase fraction. Moreover, it allowed us to compare the properties of the confined amorphous phase with those of the hypothetical unconfined one.

## 3 Results

### 3.1 P–V–T Data of the Unconfined Amorphous Phase and Crystal Phase

**Figure 3:**
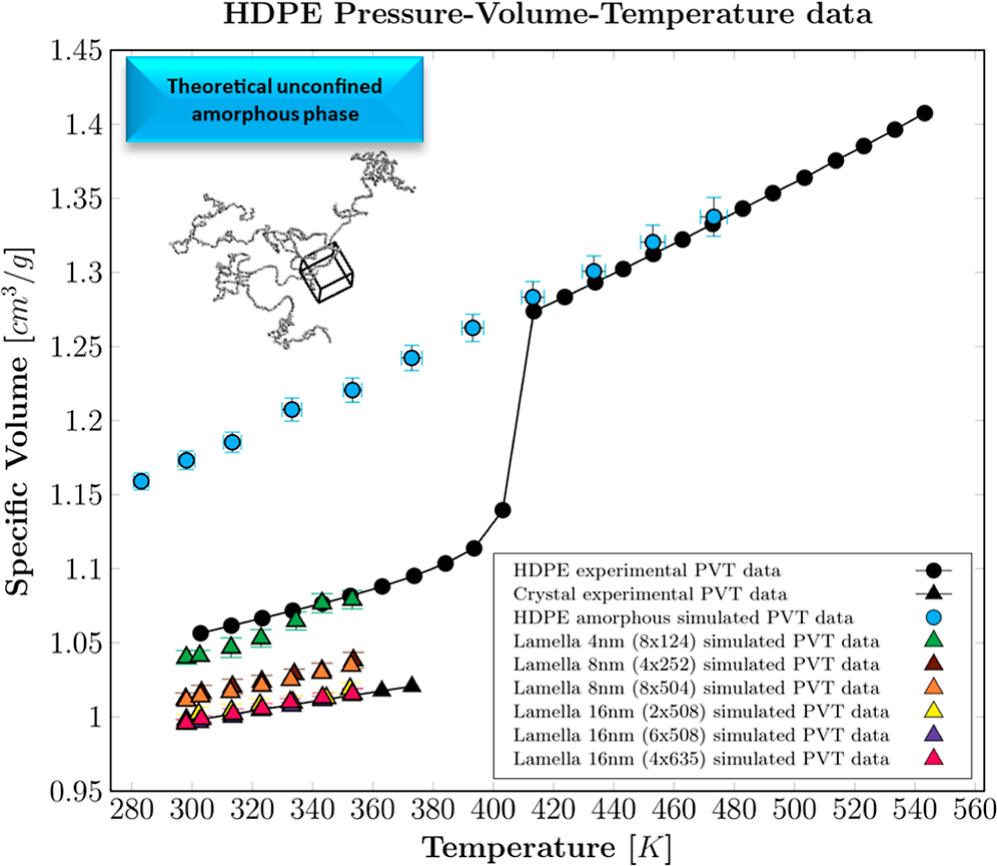
P–V–T data for HDPE at 0.1 MPa. Experimental data for the semicrystalline polymer (black dots^45^) and the crystal phase (black triangles^46^); MD data for unconfined amorphous phase (cyan dots), lamella 4 nm (8 × 124) (green triangles), lamella 8 nm (4 × 252) (brown triangles), lamella 8 nm (8 × 504) (orange triangles), lamella 16 nm (2 × 508) (yellow triangles), lamella 16 nm (6 × 508) (violet triangles), and lamella 16 nm (4 × 635) (pink triangles).

MD simulation results obtained for the unconfined amorphous phase and the set of crystalline lamellae are displayed in Figure 3 against the experimental references. Since some data overlap, numerical results are provided in Table S1 of the Supporting Information. Experimental P–V–T data of molten and semicrystalline HDPE were taken from literature,^45^ whereas the pure crystal data were estimated from experimental X-ray measurements of the thermal dilation of crystal unit cell lattice parameters at ambient pressure.^46^

An excellent match between the simulated unconfined amorphous phase and the experimental volumetric data was achieved in the molten state. Below the melting point (*T*_m_ ≅ 408 K), all-atom MD simulations cannot capture the polymer crystallization within the short simulation times considered in this work, but they represent the hypothetical behavior of the amorphous phase in the absence of crystallization (unconfined state). The specific volume of the simulated crystalline lamellar structures did not show any significant influence on the number and length of packed aligned chains, while it decreased nonlinearly with thickness, suggesting an asymptotic behavior. Such a trend is ascribed to the perturbation of the chain folds at the lamella edges, which reduces packing ability and increases the overall specific volume of the crystals. As this effect is related to the phase boundaries, it is more significant for thin lamellae, while it becomes gradually negligible for higher thicknesses. In particular, the lamellar structures with a thickness of 16 nm faithfully reproduced the crystal experimental volumetric data. Moreover, the latter is in satisfactory agreement with recent experimental findings on linear PE crystalline lamellar thickness distribution obtained using SAXS, Raman spectroscopy, and fast scanning calorimetry (10–22 nm).^47,48^ The crystalline lamella with 4 chains of 635 monomers each was selected for the further development of the semicrystalline structures, as is the 16 nm lamella that gives the best compromise between computational cost (total number of atoms) and the extent of the superficial transverse area available for the construction of tie-chains.

### 3.2 P–V–T Data of the Semicrystalline Structures

**Figure 4:**
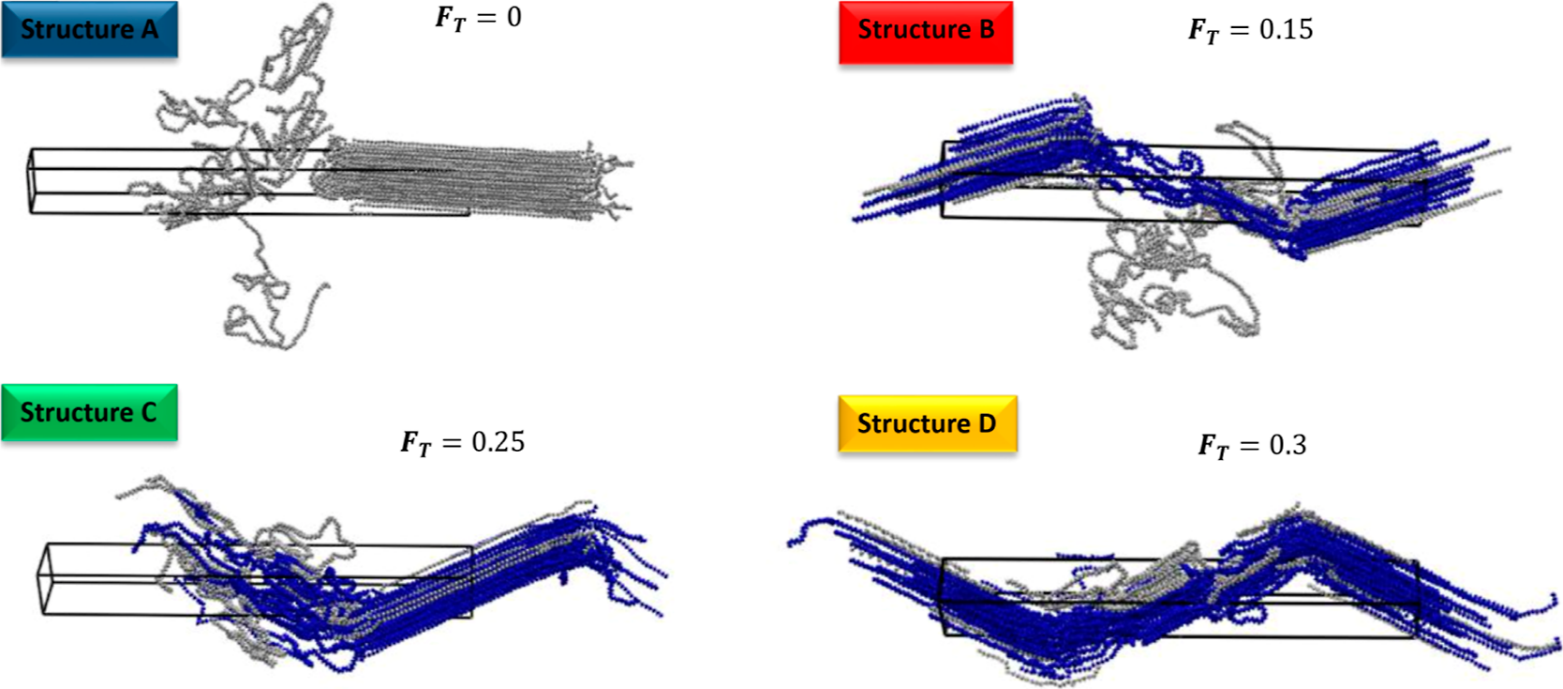
Snapshot of the simulated semicrystalline HDPE structures at the end of the equilibration stage at 298 K and 0.1 MPa. The chains involved in tie formation are depicted in blue: Structure A (no ties, *F*_T_ = 0), Structure B (6 ties, *F*_T_ = 0.15), Structure C (10 ties, *F*_T_ = 0.25), and Structure D (12 ties, *F*_T_ = 0.30).

**Figure 5:**
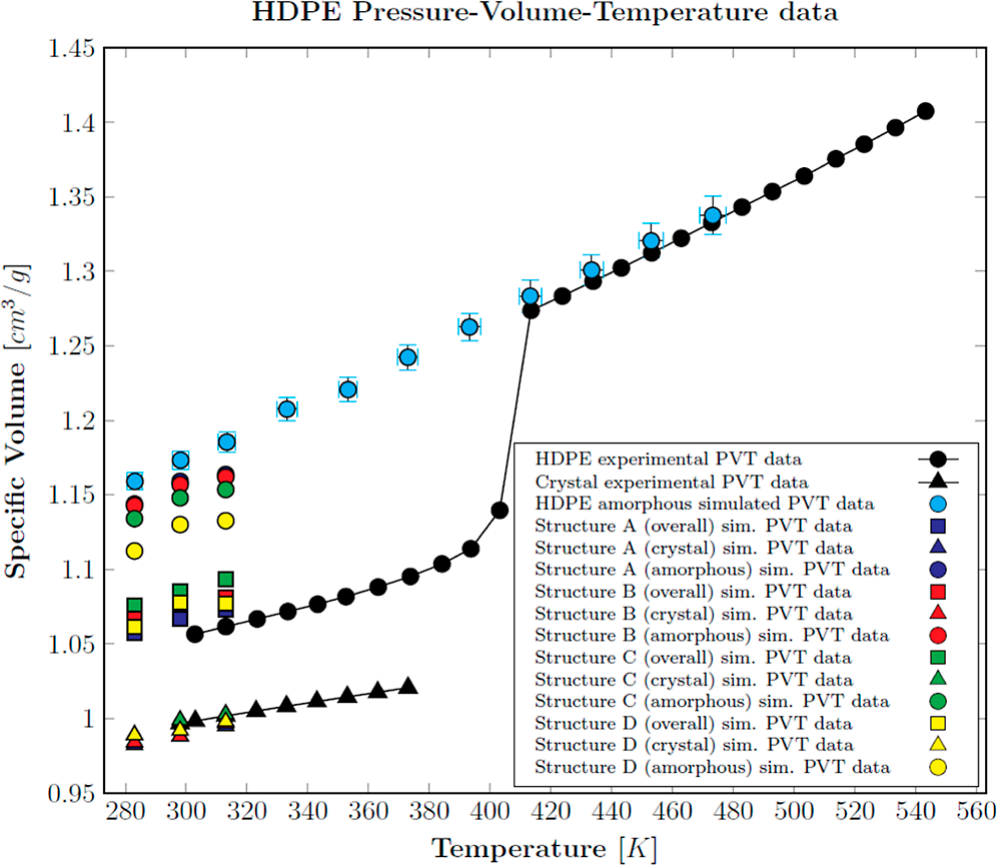
HDPE P–V–T data at 0.1 MPa for the semicrystalline structures. Experimental data for the semicrystalline material (black dots^45^) and for the crystalline phase (black triangles^46^). MD data for semicrystalline structures A (blue), B (red), C (green), and D (yellow): overall structure (squares), confined amorphous fraction (dots), and crystal fraction (triangles).

The outputs of the semicrystalline structure simulations provided the overall P–V–T data. The extraction of the confined amorphous and crystal phase fractions’ volumetric properties required the estimation of the crystal–amorphous boundaries ([*z*_–_^*am*^, *z*_+_^*am*^]) from the drastic change of *H*_*i,z*__*k*_. A snapshot of the configurations of the structures at the end of the equilibration stage under ambient conditions is provided in Figure 4, while the overall, crystal, and confined amorphous volumetric data of the simulated structures are displayed in Figure 5 against the experimental references.

Even though they are visualized in different ways, all of the structures have a single crystal per unit cell. Structures B and D are shown with the crystalline lamella split into two parts that are contiguous along the *z*-axis due to the periodic boundary conditions. On the other hand, the lamella of structures A and C is displayed as a continuous entity in which a portion falls outside the simulation box but is mirrored on the left-hand side.

The equilibrated configurations revealed the development of loose folds and chain sliding within the lamella, while the initially fully stretched tie-chains relaxed to an equilibrium elongation. Moreover, two of the 14 ties initially established in Structure D were pulled out from the crystal phase into the amorphous one during the simulation, thus reducing the area fraction of ties of the structure to 0.3.

As can be appreciated from the graph, the crystal volumetric data were all in line with the experimental reference with minimal deviations. On the other hand, as expected, the confined amorphous P–V–T data did not retrace the theoretically unconfined amorphous ones but rather shifted toward greater densities. Interestingly, the bare crystal confinement (Structure A) triggered a substantial decrease of the amorphous matrix free volume, an effect that became more significant as the fraction of tie-chains increased. The latter output must be intended as a direct consequence of the modeled crystal–amorphous interplay since all the simulations had the same initial amorphous stack density. Finally, the location of the semicrystalline P–V–T data was in satisfactory agreement with the experimental reference; the incomplete overlap can be reasonably attributed to the discrepancy in the degree of crystallinity between the simulated structures and the experimental sample, which was not available.

### 3.3 Sorption Coefficient Results

**Figure 6:**
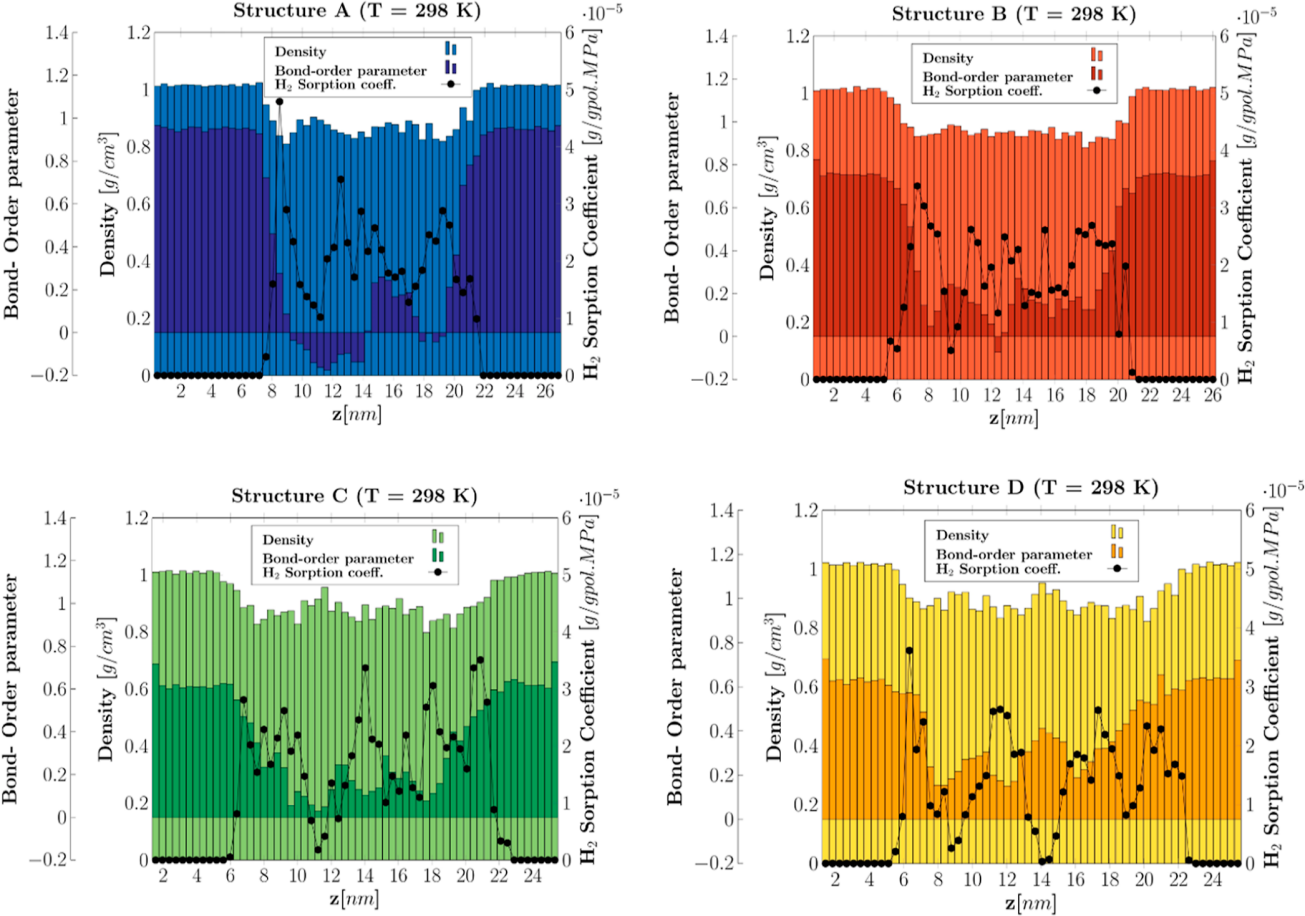
Profiles of density, bond order parameter, and hydrogen sorption coefficient along the *z*-axis for the semicrystalline structures at 298 K and 0.1 MPa. Density: bars (Structure A: blue, Structure B: light red, Structure C: light green, and Structure D: light yellow). Bond-order parameter: bars (Structure A, dark blue; Structure B, dark red; Structure C, dark green; and Structure D, orange). Henry’s law coefficient: black dots.

The analysis of the discretized simulation boxes along the *z*-axis allowed for the estimation of the tight relation among the density, bond-order orientation parameter, and sorption coefficients for the different structures. For the sake of brevity, only the results at 298 K are reported in Figure 6.

As displayed in the charts, a close connection between the density, orientation, and sorption coefficient was found. The Henry’s constant of the crystal phase was evaluated to be at least 10 orders of magnitude greater than the amorphous one, hence largely confirming the hypothesis of negligible sorption in HDPE crystal domains even for the small hydrogen molecule. Although recent molecular simulation results have shown the ability of hydrogen molecules to perform fast, long jumps through the ordered aligned chains of crystalline HDPE,^49^ the resulting diffusion coefficient is not sufficient to counterbalance the effect of the extremely low sorption capacity found here; therefore, the assumption of impermeable crystal domains is also expected to be valid. The presence of a narrow crystal–amorphous interphase (around 10–13 Å) was coherently identified by all three analyses and that is in accordance with other findings in the literature.^50,51^ Moreover, the simulation analysis evidenced that, when the fraction of ties increases, they tend to form semiordered packed clusters in the amorphous phase bulk and that reflects in significant drops of the sorption capacity. The wavy trend of the sorption coefficient in the amorphous phase is due to the density fluctuations in each slice, which depend on the number of slices in use. However, the discretization was mainly done to identify the crystal–amorphous transition, while a statistically meaningful correlation can only be established between the average sorption coefficient of the amorphous stack (eq 11) and its average density.

While there exists a tight correlation between density and hydrogen sorption coefficient, the bond-order orientation has no direct influence on sorption but serves mainly as an additional parameter to evidence the crystal–amorphous boundaries. Moreover, from its average value in the crystal stack, it is possible to determine the tilt angle, which, in turn, shows a dependence on the surface fraction of tie-chains and consequently on the average density and sorption coefficient of the confined amorphous phase, as will be reported in the next section. The results at ambient conditions are summarized in Table 2.

**Table 2:** Main Features of the Structures Simulated in This Work Are Shown at 298 K and 0.1 MPa

*T* = 298 K *p* = 0.1 MPa		unconfined amorphous phase	Structure A	Structure B	Structure C	Structure D
*F*_T_			0	0.15	0.25	0.3
density(g/cm^3^)	overall		0.937	0.926	0.922	0.928
	amorphous	0.852	0.863	0.864	0.871	0.885
	crystal		1.012	1.012	1.001	1.002
ω_cr_		0	0.540	0.458	0.426	0.397
θ_tilt_			10°	24°	30°	30°

The data in Table 2 show that upon increasing the surface fraction of ties, the average amorphous matrix density increases, and the final degree of crystallinity ω_cr_ decreases. The trade-off between the area fraction of ties and the crystal amount or thickness agrees with both statistical and mechanical approaches which estimated the tie-chain fraction in samples with different degrees of crystallinity.^17,52,53^ This trade-off indicates that a negligible fraction of the tie-chains should be expected for high crystallinity samples (slowly cooled), whereas significant fractions are assumed to be present in lower crystallinity samples (quenched materials), a correlation that may be useful to correlate the material parameters to the processing conditions.

On the other hand, a more pronounced lamella tilt angle, θ_tilt_, was observed for a higher fraction of ties (up to 30°) and that is, interestingly, in agreement with the conclusions drawn by Gautam et al. who studied the chain population distribution resulting from off-lattice Monte Carlo simulations on systems at fixed chain tilts.^54^ In their study, they interpreted lamella tilting as a mechanism for dissipating the density and bond-order flux across the crystal–amorphous boundary, and they found that a tilt angle equal to 34.4° corresponded to the least interfacial energy. Moreover, in a recent article, Kanomi et al.^55^ analyzed the experimental lamella tilt angle distribution in semicrystalline polyethylene through the electron-diffraction-based imaging technique and showed that isolated lamellae exhibit a high frequency of tilt angles close to 30°, while stacked lamellae showed lower values around 8°. The modeling results obtained in this work, which provide a useful direct relation between the value of the tilt angle and the expected surface fraction of tie-chains, may be used for the prediction of several material features based on the experimental determination of the tilt angle.

### 3.4 Sorption Coefficient in the Amorphous Phase: Unconfined vs. Confined

Comparing sorption coefficients in semicrystalline HDPE samples having different values of degree of crystallinity might be misleading due to the demonstrated negligible solubility of hydrogen in the crystal fraction. Therefore, hydrogen sorption coefficients in the semicrystalline material (*S*_H2_) available in the literature were referred to as the solely confined amorphous phase fraction (*S*_H2_^*am*^) using the following additive rule, if the corresponding degree of crystallinity was indicated
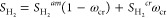
12
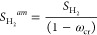
13

where *S*_H2_^*cr*^ represents the sorption coefficient in the crystal phase, which was evaluated to be equal to zero, thus providing the relation reported in eq 13.

The aforementioned comparison is summarized in Table 3 together with other details of the different experimental samples, such as the mass degree of crystallinity and the density of the amorphous phase. The uncertainty of the simulated sorption coefficient value was estimated by the block averaging method, computing the standard deviation of 10 blocks (100 frames each).

**Table 3:** Hydrogen Sorption Coefficient in the HDPE Amorphous Phase Fraction: Comparison between the Widom Test Results of the Simulated Structures and Literature Data

hydrogen sorption coefficient in the HDPE amorphous phase fraction						
		*S*_H2_^*am*^ [10^–5^gH_2_/(gHDPE am. MPa)]				references
*T* (K)	*P* (MPa)	Structure A	Structure B	Structure C	Structure D	this work
		**ρ** = 0.937 (g/cm^3^)	**ρ** = 0.926 (g/cm^3^)	**ρ** = 0.922 (g/cm^3^)	**ρ** = 0.928 (g/cm^3^)	
		ω_*cr*_ = 0.540	ω_*cr*_ = 0.458	ω_*cr*_ = 0.426	ω_*cr*_ = 0.397	
		(*F*_T_ = 0)	(*F*_T_ = 0.15)	(*F*_*T*_ = 0.25)	(*F*_T_ = 0.30)	
283	0.1	**1.99** ± 0.18	**1.88** ± 0.16	**1.82** ± 0.16	**1.61** ± 0.2	
298	0.1	**2.01** ± 0.15	**1.84** ± 0.08	**1.71** ± 0.09	**1.48** ± 0.15	
313	0.1	**2.15** ± 0.22	**1.81** ± 0.16	**1.56** ± 0.14	**1.49** ± 0.15	

*T* (K)	*P* (MPa)	**method**	**ρ**(g/cm^3^)	ω_cr_	*S*_*H*2_^*am*^	references
293		*permeation test*		0.5–0.6 (assumed)	**1.81–2.26**	Humpenoder^56^
						Barth et al.^57^
303	10–90	*permeation test*	0.945	0.53–0.57	**2.95–2.90**	Fujiwara et al.^58^
300	70	*molecular modeling*		fully amorphous model	**1.36**	Voyiatzis et al.^59^
300	15–100	*molecular modeling*	0.950	0.4953	**4.46–2.34**	Zhao et al.^60^

The experimental findings from Fujiwara et al. highlight a weak dependence of the hydrogen sorption coefficient with pressure (1.7% decrease passing from 10 to 90 MPa), hence suggesting a reliable comparison between results obtained at different pressures.

**Figure 7:**
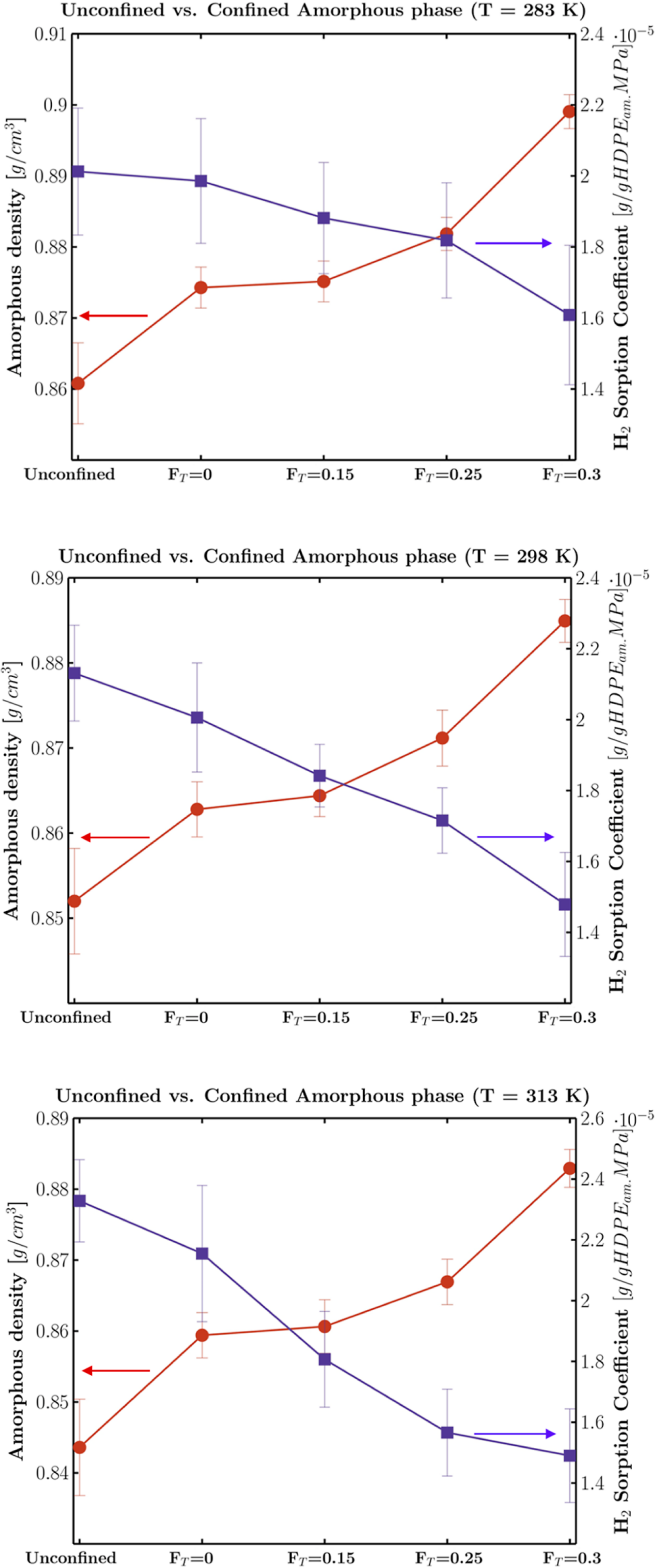
Effect of the crystal confinement and/or area fraction of tie-chains (*F*_T_) on the amorphous phase fraction density (brick red dots) and hydrogen sorption coefficient (violet squares) at *T* = 283, 298, and 313 K.

**Figure 8:**
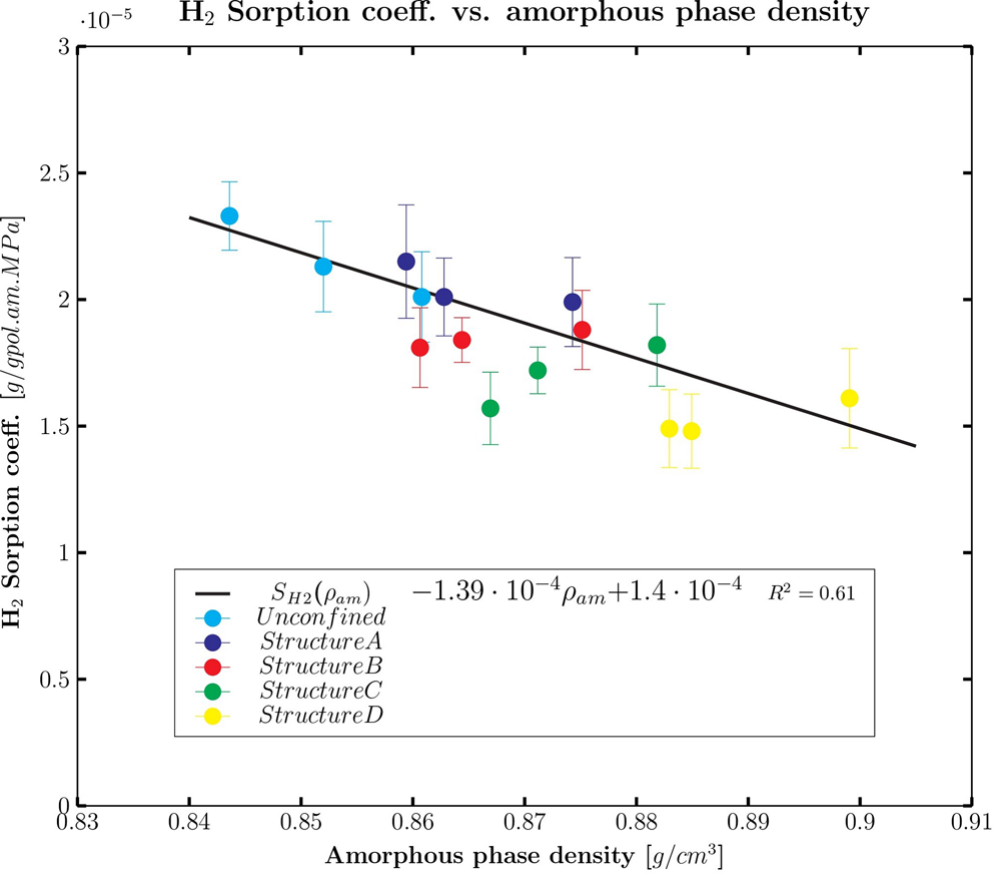
Simulated sorption coefficient of hydrogen in the amorphous phase fraction against the amorphous phase density for all simulated structures and temperatures: simulated data (dots) and linear fitting (black line).

The sorption coefficients obtained in this work were in agreement with the experimental results of permeation tests.^56−58^ The effect of the confinement or the surface fraction of tie-chains on the amorphous phase density and relative hydrogen sorption coefficient is displayed in Figure 7. The uncertainty of the simulated amorphous density was estimated by the block averaging method. In addition, the trend of the whole set of simulated sorption coefficient data against the corresponding amorphous phase density is reported in Figure 8.

As already mentioned in Section 2.3, at each temperature, a progressive increase in the density is observed, going from the unconfined amorphous state to the confined state having the greatest fraction of ties, and that increase is accompanied by an opposite trend of the estimated sorption coefficient. Interestingly, a significant drop of the sorption capacity is observed just because of the confinement between crystal layers. The addition of a progressively higher fraction of ties causes a further increase of the density and a consequent reduction of the sorption capacity so that the maximum relative drop of the amorphous phase sorption coefficient with respect to the theoretically unconfined state reaches 24, 32, and 38% at *T* = 283, 298, and 313 K, respectively.

In Figure 8, the sorption coefficient was correlated with the amorphous density: it can be seen that a linear trend exists between these two quantities regardless of the type of confinement felt by the amorphous phase and the temperature. This is an additional correlation resulting from the simulations carried out in this work, which can be used for rule-of-thumb estimates of hydrogen sorption in different HDPE samples of known density and crystallinity.

The dependence of the sorption coefficient on temperature is rather weak in all cases (see Figure S2 in the Supporting Information), and the differences due to temperature are generally within the uncertainty of the simulated values, preventing any conclusion about the sign of the enthalpy of sorption.

## 4 Conclusions

In this work, we used an all-atom approach to simulate the volumetric and hydrogen sorption properties of semicrystalline HDPE samples with increasing connectivity between the amorphous and crystal phases. The molecular modeling approach illustrated in this work allowed the hypothesis of negligible hydrogen sorption in the HDPE crystal phase to be confirmed and provided direct quantitative information on the morphological perturbation induced by the latter on the confined amorphous phase fraction. A significant increase in the amorphous phase density with respect to its theoretical unconfined state was observed just because of the confinement between crystals. At higher fractions of ties, the constraint effect became more and more important, and a contextual reduction in the mass degree of crystallinity and an increase in the lamella tilting were observed. The values of the sorption coefficients in the amorphous phase fractions were in good agreement with experimental references available in the literature and reflected the trend of the density.

The modeling results allowed us to relate a series of crystal–amorphous phase intercalations, which could potentially reproduce experimental samples obtained with different processing conditions, to the resulting amorphous density and hydrogen sorption capacity. The knowledge of the actual area fraction of ties within a sample of a given thermal history is necessary to use these modeling results as a direct predictive tool for the evaluation of the gas sorption capacity. Moreover, the outputs of the present work evidenced that, due to its sensitivity to the area fraction of ties, the experimental value of the lamella tilt angle might figure as a promising candidate for its assessment. Together with simulated diffusion coefficients, which will be the object of future work, these results will allow us to estimate the effect of the semicrystalline microstructure on the hydrogen permeability in HDPE.

## Supporting Information Available

Simulated P–V–T data at *P* = 0.1 MPa obtained for the different crystalline lamellae built in this work against experimental P–V–T data and hydrogen sorption coefficient in the amorphous phase fraction as a function of temperature for all the simulated structures plus Arrhenius’ law fitting (PDF)
