# Supplementary material for: Molecular Simulations of Hydrogen Sorption in Semicrystalline High-Density Polyethylene: The Impact of the Surface Fraction of Tie-Chains
Source: J Phys Chem B. 2024 Mar 7;128(11):2799–810. doi: 10.1021/acs.jpcb.3c07705 (PMC10961721; doi:10.1021/acs.jpcb.3c07705)
Supplement: Supplementary file 1 — jp3c07705_si_001.pdf [file jp3c07705_si_001.pdf]

Molecular Simulations of Hydrogen Sorption in Semi-crystalline High-Density Polyethylene: The Impact of the Surface Fraction of Tie-chains

Omar Atiq [a,c], *Eleonora Ricci* [b,c], *Marco Giacinti Baschetti* [a,c], *Maria Grazia De Angelis* [a,b,c*]

[a] Department of Civil, Chemical, Environmental and Material Engineering, (DICAM), Alma Mater Studiorum – Università di Bologna, via Terracini 28, 40131 Bologna, Italy.

[b] Institute for Materials and Processes, School of Engineering, University of Edinburgh, Sanderson Building, Robert Stevenson Road, EH9 3FB, Scotland, UK.

[c] DPI, P.O. Box 902, 5600 AX Eindhoven, the Netherlands.

*Corresponding Author; e-mail: Grazia.deangelis@ed.ac.uk

Table S1 Simulated P-V-T data at P = 0.1 MPa obtained for the different crystalline lamellae built in this work against experimental P-V-T data [46].

**Crystalline Lamellar Structures**												**Experimental data [43]**	
**4nm_8x124**		**8nm_4x252**		**8nm_8x504**		**16nm_2x508**		**16nm_6x508**		**16nm_4x635**			
$\boldsymbol{T}$(°C)	$\hat{\boldsymbol{V}}\left(\frac{cm^3}{g}\right)$	$\boldsymbol{T}$(°C)	$\hat{\boldsymbol{V}}\left(\frac{cm^3}{g}\right)$	$\boldsymbol{T}$(°C)	$\hat{\boldsymbol{V}}\left(\frac{cm^3}{g}\right)$	$\boldsymbol{T}$(°C)	$\hat{\boldsymbol{V}}\left(\frac{cm^3}{g}\right)$	$\boldsymbol{T}$(°C)	$\hat{\boldsymbol{V}}\left(\frac{cm^3}{g}\right)$	$\boldsymbol{T}$(°C)	$\hat{\boldsymbol{V}}\left(\frac{cm^3}{g}\right)$	$\boldsymbol{T}$(°C)	$\hat{\boldsymbol{V}}\left(\frac{cm^3}{g}\right)$
24.98	1.040	24.77	1.012	24.98	1.011	24.98	0.998	25.20	0.996	24.90	0.996	25	0.997
29.43	1.041	29.98	1.016	29.63	1.014	29.07	1.002	29.16	0.997	29.98	0.999	30	0.998
39.82	1.047	40.29	1.020	39.61	1.017	39.98	1.004	40.32	1.000	40.49	1.002	40	1.002
49.76	1.053	50.01	1.024	50.00	1.021	49.42	1.008	49.67	1.005	50.15	1.006	50	1.005
61.36	1.065	60.79	1.029	59.62	1.025	60.30	1.010	59.91	1.008	59.47	1.010	60	1.008
70.04	1.077	69.99	1.031	69.84	1.030	71.46	1.013	70.17	1.011	70.17	1.013	70	1.012
80.05	1.079	80.41	1.038	79.64	1.035	79.33	1.019	80.10	1.016	80.10	1.015	80	1.015

Molecular Simulations of Hydrogen Sorption in Semi-crystalline High-Density Polyethylene: The Impact of the Surface Fraction of Tie-chains

Omar Atiq [a,c], *Eleonora Ricci* [b,c], *Marco Giacinti Baschetti* [a,c], *Maria Grazia De Angelis* [a,b,c*]

[a] Department of Civil, Chemical, Environmental and Material Engineering, (DICAM), Alma Mater Studiorum – Università di Bologna, via Terracini 28, 40131 Bologna, Italy.

[b] Institute for Materials and Processes, School of Engineering, University of Edinburgh, Sanderson Building, Robert Stevenson Road, EH9 3FB, Scotland, UK.

[c] Dutch Polymer Institute (DPI), P.O. Box 902, 5600 AX Eindhoven, the Netherlands.

*Corresponding Author; e-mail: Grazia.deangelis@ed.ac.uk

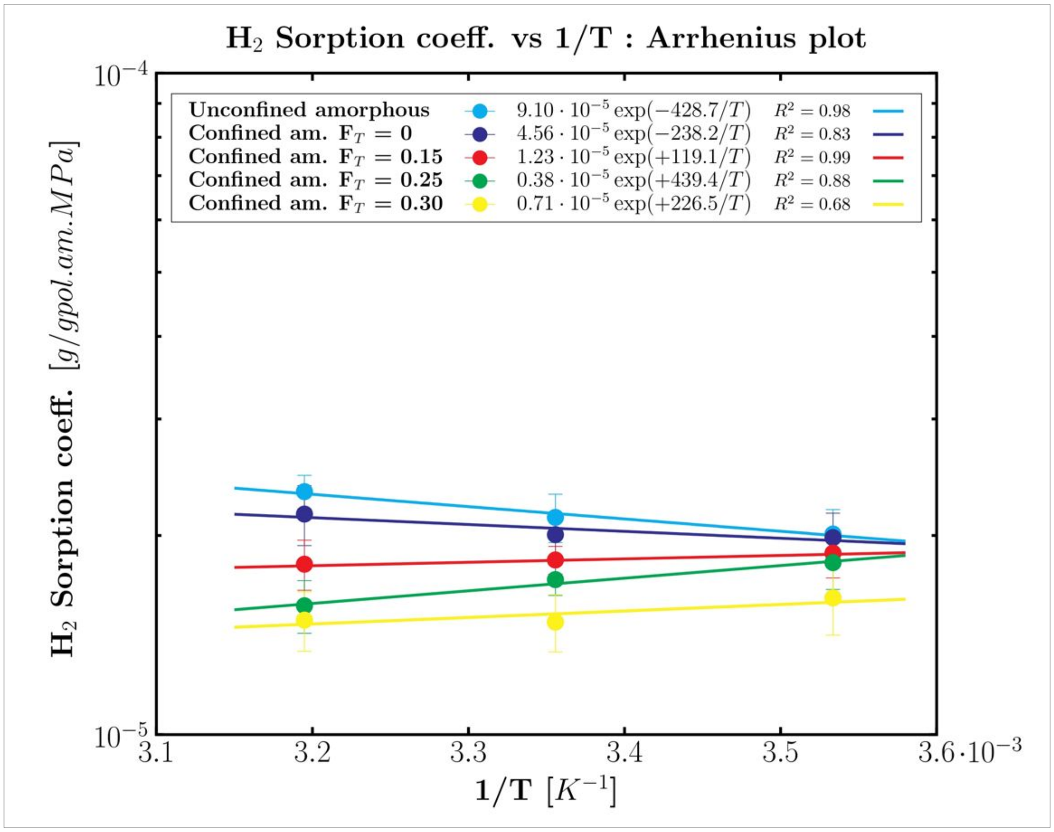

Figure S1 Hydrogen sorption coefficient in the amorphous phase fraction as a function of temperature for all the simulated structures plus Arrhenius' law fitting (continuous lines):

Theoretical unconfined amorphous phase (cyan dots). Confined amorphous phases of Structure A, B, C and D (blue, red, green, and yellow dots).
